# Recommendations for the use of bioresorbable vascular scaffolds in percutaneous coronary interventions: 2017 revision

**DOI:** 10.1007/s12471-017-1014-z

**Published:** 2017-06-22

**Authors:** B. Everaert, J. J. Wykrzykowska, J. Koolen, P. van der Harst, P. den Heijer, J. P. Henriques, R. van der Schaaf, B. de Smet, S. H. Hofma, R. Diletti, A. Weevers, J. Hoorntje, P. Smits, R. J. van Geuns

**Affiliations:** 1 Thoraxcenter, Erasmus Medical Centre, Rotterdam, The Netherlands; 2 Academic Medical Center, Amsterdam, The Netherlands; 3 Catharina Hospital, Eindhoven, The Netherlands; 4 University Medical Center, University of Groningen, Groningen, The Netherlands; 5 Amphia Hospital, Breda, The Netherlands; 6 Onze Lieve Vrouwe Gasthuis, Amsterdam, The Netherlands; 7 Meander Medical Center, Amersfoort, The Netherlands; 8 Medical Center, Leeuwarden, The Netherlands; 9 Albert Schweitzer Hospital, Dordrecht, The Netherlands; 10 Maastricht University Medical Center, Maastricht, The Netherlands; 11 Maasstad Hospital, Rotterdam, The Netherlands; 12 Monica Hospital, Antwerp, Belgium

**Keywords:** Bioresorbable vascular scaffold, Percutaneous coronary intervention, Absorb BVS

## Abstract

**Background:**

To eliminate some of the potential late limitations of permanent metallic stents, the bioresorbable coronary stents or ‘bioresorbable vascular scaffolds’ (BVS) have been developed.

**Methods:**

We reviewed all currently available clinical data on BVS implantation.

**Results:**

Since the 2015 position statement on the appropriateness of BVS in percutaneous coronary interventions, several large randomised trials have been presented. These have demonstrated that achieving adequate 1 and 2 year outcomes with these first-generation BVS is not straightforward. These first adequately powered studies in non-complex lesions showed worse results if standard implantation techniques were used for these relatively thick scaffolds. Post-hoc analyses hypothesise that outcomes similar to current drug-eluting stents are still possible if aggressive lesion preparation, adequate sizing and high-pressure postdilatation are implemented rigorously. As long as this has not been confirmed in prospective studies the usage should be restricted to experienced centres with continuous outcome monitoring. For more complex lesions, results are even more disappointing and usage should be discouraged. When developed, newer generation scaffolds with thinner struts or faster resorption rates are expected to improve outcomes. In the meantime prolonged dual antiplatelet therapy (DAPT, beyond one year) is recommended in an individualised approach for patients treated with current generation BVS.

**Conclusion:**

The new 2017 recommendations downgrade and limit the use of the current BVS to experienced centres within dedicated registries using the updated implantation protocol and advise the prolonged usage of DAPT. In line with these recommendations the manufacturer does not supply devices to the hospitals without such registries in place.

## Introduction

Drug-eluting stents (DES) are widely used and considered first choice devices in percutaneous coronary interventions (PCI) to treat ischaemic coronary artery disease [1]. These permanent implants, however, do not have any residual function after vascular healing following PCI. Beyond the initial healing period, metallic stents may induce new problems, resulting in an average reintervention rate of 2% per year [2]. To eliminate some of the potential late limitations of permanent metallic DES, such as impaired vasomotion, hampered endothelial function, reduced potential for vessel remodelling and interference with future non-invasive imaging (cardiac computed tomography or magnetic resonance imaging) or treatment modalities (re-PCI or coronary artery bypass grafting), bioresorbable coronary stents or ‘bioresorbable vascular scaffolds’ (BVS) have been developed. As clinical evidence is accumulating, we have updated the 2015 Dutch recommendations on the use of BVS in PCI [3].

## Lesion selection

The short-term efficacy of BVS in patients with non-complex coronary lesions has been investigated and reported in four large independent randomised trials (ABSORB II, ABSORB China, ABSORB Japan and ABSORB III [4–7]). The characteristics of these ‘Absorb 2/3-like lesions’ are summarised in Table 1.

**Table 1:** BVS Absorb 2/3-like lesions

Absorb 2/3-like lesions	Exclusion
De novo lesions	Left main
Diameter 2.3–3.8 mm	Arterial or venous grafts
Maximum length 28 mm	In-stent restenosis
One BVS scaffold overlap	Chronic total occlusion
Maximum 2 lesions	Ostial lesions
Stable, unstable or silent ischaemia	Bifurcation lesions with side branches ≥2 mm diameter
–	Excessive calcification
–	High tortuosity
–	Visible thrombus
–	(N)STEMI
–	LVEF <30%

At 1 year, Absorb BVS (Abbott Vascular, Santa Clara, CA) were demonstrated to be non-inferior to Abbott’s Xience DES regarding the combined and individual clinical endpoints of death, myocardial infarction (MI), and target lesion failure/revascularisation. These results were confirmed in a meta-analysis of randomised clinical trial data at 1 year, comprising 3389 patients randomised in a 2:1 fashion to Absorb BVS and Xience DES, respectively [8]. However, a signal of higher incidence of target vessel MI was reported, partly due to a non-significant nominal increase in device thrombosis (hazard ration (HR) 2.09). In the longer term, the 5‑year results of the ABSORB cohort B trial were encouraging, showing late lumen stability and the restoration of vasomotor function, together with low restenosis and major adverse cardiac event (MACE) rates in relatively simple stenotic lesions [9]. This was in line with expectations regarding a potential long-term physiological benefit after full bioresorption of scaffolds over permanent metallic DES. However, after 3 years, the ABSORB II study did not report any superiority in vasomotor reactivity of BVS over DES [10]. Moreover, the device-oriented composite endpoint was significantly worse in the Absorb BVS subgroup (HR 2.17), mainly due an increase in target vessel MI (6% vs. 1%; *p* = 0.011).

Furthermore, also the 2 year results from the ABSORB III study were reported [11] and the rates of target lesion failure were significantly higher with BVS than DES (11.0% vs. 7.9%, *p* = 0.03), mainly driven by an increase in target vessel MI, which was partly due to an increase in the incidence of scaffold thrombosis (ScT).

In the initial ABSORB trials, the implantation technique was reported to be suboptimal according to current BVS implantation standards. In a pooled analysis (ABSORB II, ABSORB China, ABSORB Japan and ABSORB III and ABSORB Extend) optimal sizing, optimal sizing/predilatation and optimal sizing/pre- and post-dilatation was only achieved in about 81.6%, 59.2% and 12.4% of patients, respectively [12]. In particular optimal sizing avoiding implantation in vessels with a reference vessel diameter (RVD) by quantitative coronary analysis (QCA) of <2.25 mm or >3.5 mm reduced both 2 year target lesion failure (12.1% vs. 7.6%, *p* = 0.0006) and stent thrombosis (2.6% vs. 1.5%, *p* = ns) [13]. In a blinded, pooled interim analysis of ABSORB IV, the rate of ScT at 30 days and after 1 year was lower compared with ABSORB III (0.3% vs. 1.0% and 0.5% vs. 1.3%, respectively), which was attributed to higher adherence to improved implantation techniques, in particular avoidance of implantation in vessels with an RVD <2.25 (4% vs. 19%, respectively) and applying proper post-dilatation (83% vs. 66%, respectively). Still we will have to wait for the completion of this trial to see the unblinded data.

Given the lack of clear benefit on mid-term outcomes compared with contemporary DES with repeated signals of safety issues regarding early, late and possibly very late stent thrombosis, we have to downgrade the level of recommendation for ‘Absorb 2/3-like lesions’ from ‘appropriate’ to ‘potentially appropriate’ and restricted to experienced centres with continuous safety monitoring within dedicated registries using the most recent implantation technique (as described in the instructions for use of the device). Patient should be well informed on the potential risks of the therapy and the only hypothetical long-term benefit.

For patients with more complex lesions, who were excluded from the aforementioned Absorb 2/3 studies, the mid-term outcome data of some medium-sized studies on more complex, real-world lesions and indications have been reported. For instance, the BVS Expand Registry, a single-centre registry including a more complex patient and lesion subset, showed a clinical device success of 97.3% and a MACE rate of 6.8% at 18 months [14]. Secondly, from a large real-world BVS registry (GHOST-EU) the 1‑year clinical result data were compared with propensity-matched patients from a large post-marketing DES registry (Xience V USA). No differences in target lesion failure, target vessel MI or stent/scaffold thrombosis were noticed [15].

Recently, the Amsterdam Investigator-initiateD Absorb Strategy all-comers trial (AIDA) trial, an investigator-initiated, randomised non-inferiority trial in the context of routine clinical practice, was published early because of safety concerns raised by the study data and safety monitoring board (median duration of follow-up of 707 days) [16]. This study included much more complex patients compared with the company initiated Absorb phase 3 studies requirements for approval. In AIDA, 54% of the patients presented with acute coronary syndrome (ACS), which was not allowed in the Absorb studies. A large number of complex lesions were included (5% bifurcations, 4% chronic total occlusions (CTO), 5.6% ostial and 30% moderately or severely calcified) with on average longer lesions and more scaffolds per patient and per lesion. Still there was no significant difference regarding the primary endpoint of target vessel failure at 2 years (11.7% vs. 10.7%) nor in the secondary endpoints of target vessel and target lesion revascularisation (8.7% vs. 7.5%, 7.0% vs. 5.2%, respectively). Most importantly, there was a highly significant difference in the rates of definite and probable ScT at 2 years (3.5% with BVS vs. 0.9% with metallic DES, *p* < 0.001). This was reflected in a higher rate of target vessel MI (5.5% vs. 3.2%, *p* = 0.04). A more detailed analysis showed, besides an increase in early and late ScT, also the occurrence of very late ScT. For this reason, the data and safety monitoring board recommended early reporting of the study to inform the patients and physicians. Concerning the implantation technique, the use of postdilatation was higher compared with previous studies (74%) but not yet at the current target level and as in many other studies, small vessels (reference vessel diameter ≤2.25 mm by QCA) accounted for almost 20% of the lesions. Vessel size of ≤2.25 mm, adequate device sizing or postdilatation did not seem to be not associated with the occurrence of ScT; however, the relatively small sample size (31 ScT cases) might have limited this analysis. This contrasts with the findings from a case-control analysis of 105 ScTs occurring in randomised controlled trials (RCTs) and large registries (>200 BVS-treated patients) with at least 12 months of follow-up [17], in which early dual antiplatelet therapy (DAPT) cessation, no-postdilatation and RVD <2.4 mm were identified as predictors for ScT.

The GHOST-EU registry included 302 bifurcation lesions. The rate of target lesion failure with BVS was acceptable (6.4% at 360 days). However, the rates of ScT were elevated (2.5%) [18]. For long coronary lesions (≥60 mm), target lesion failure rate with BVS was substantially higher (14.3% compared with 4.8% and 4.5% for lesions <30 mm or between 30 and 60 mm, respectively), mainly because of an increase in MI (including ScT (3.8%)) and clinically driven target lesion revascularisation [19]. Of note, scaffold overlapping did not appear to have a negative impact on a composite endpoint of all-cause death, any MI and any repeated revascularisation as well as on the rate of early or late ScT [20]. Concerning BVS implantation in ACS patients (ST-segment elevation myocardial infarction (STEMI), non-STEMI, unstable angina) the rates of patient- and device-oriented endpoints, as well as ScT, were significantly elevated compared with non-ACS patients [21]. One multicentre, single-blinded, RCT reported on BVS use in primary PCI in patients with STEMI. In the TROFI II study, arterial healing with BVS implantation was comparable to DES and nearly complete at 6 months and target lesion revascularisation at 6 months was low in both study arms (1.1% vs. 0%) [22]. Regarding CTOs, a recent study on complex CTO (all lesions with J‑CTO score >2, mean 2.61) reported procedural success rates of 97.1% and favourable mid-term results at up to 6 months with 3 MACE in 105 patients (2.9%) [23]. We have now included this group of lesions in the general more complex group. Still, longer follow-up is needed to draw more definitive conclusions on BVS use in both STEMI and CTO lesions.

In summary, the new data on BVS use in more complex lesions for up to 2 years is more concerning compared with our previous analysis and we changed the previous advice from probably appropriate to discouraged for routine clinical practice.

The data on ‘highly’ complex lesions, such as two scaffold bifurcations, severely calcified lesions and aorta-ostial lesions, however, are still premature or not in favour of BVS use. As such, for these highly complex lesions the use of BVS is currently not supported by expert opinion.

Furthermore, for two subsets of lesions, namely arterial or venous grafts and in-stent restenosis, the current Absorb BVS label (de novo lesions in native vessels) does not apply and the off-label use of BVS should be extraordinary.

A final – technical – limitation is the overexpansion capabilities of the Absorb BVS that is currently restricted to 0.5 mm based on the recommendation of the manufacturer. As the largest commercially available Absorb BVS is 3.5 mm at nominal pressure, vessels with a diameter above 4.0 mm should not be targeted because of the risk of extensive malapposition. We do not support the implantation of BVS in lesions with an RVD below 2.25 mm (as measured by QCA) as the outcome in this lesion subgroup is significantly worse as reported by the Absorb III trial investigators (target lesion failure: 12.9% (Absorb BVS) vs. 8.3% (Xience DES)) [5]. Another caveat is ostial coronary lesions, as was suggested by data from the GHOST-EU registry [24]. The composite endpoint of a combination of cardiovascular death, target vessel MI or target lesion revascularisation was significantly higher in ostial lesions (12.6% vs. 4.6%) as was the incidence of ScT (4.9% vs. 2.0%). The use of BVS in this group is not supported by sufficient data, and the authors of this consensus statement advise to avoid use in these lesions until more data are available.

Table 2 provides the advice of the authors on lesion characteristics used for identification of possible target lesions as descripted above.

**Table 2:** Lesion selection

Potentially appropriate^a^ using optimal implantation technique (PSP)	Absorb 2/3-like lesions: ‘de novo’ lesions, max. length 28 mm, one stent overlap, max. 2 lesions, RVD >2.25 mm on QCA
Discouraged for routine clinical practice^a^	ACS patients, including STEMI Long lesions (>28 mm, <60mm ) Moderately to severely calcified lesions with proper lesion preparation (diameter stenosis <40% after pre-dilatation) Provisional bifurcation treatment (including fenestration of the side branch) Non-complex CTO (J-CTO score <2) Complex CTO (J-CTO score ≥2)
Use not supported by data or expert opinion	Very long lesions (≥60 mm) Ostial coronary lesions Severely calcified lesions with failure to prepare properly Bifurcation lesions requiring a two scaffold approach
Not recommended	RVD <2.5 (2.25 mm on QCA) In-stent restenosis^b^ Arterial and venous grafts^b^ Vessel >4.0 mm in diameter^b^

*ACS* acute coronary syndrome, *CTO* chronic total occlusion, *PSP* pre-dilate, size properly and post-dilate, *QCA* quantitative coronary analysis, *RVD* reference vessel diameter, *STEMI* ST-elevation myocardial infarction

^a^Use restricted to dedicated BVS clinical trial/registries

^b^Off-label use

## Patient selection

As bioresorbable scaffolds resolve 2–3 years after implantation, improvement in patient outcomes in comparison with permanent metallic structures, if present, will probably be most evident in patients whose life expectancy exceeds those first years of implantation.

It is therefore essential to appropriately select patients in which BVS may yield the highest advantage on long-time clinical outcome compared with DES. The ideal BVS candidate is a young first time presenter with a good life expectancy (>5 years). On the other hand, patients above 80 years, patients with severe renal failure or on dialysis and patients who are in cardiogenic shock at the time of the implantation only have a limited life expectancy [25] and therefore the potential for a long-term benefit of BVS compared with DES is severely hampered. Other patient-related conditions, such as diabetes mellitus, body mass index >40, left ventricular ejection fraction <40%, previous stroke, peripheral artery disease and chronic obstructive pulmonary disease, also have a negative impact on patient’s life expectancy and should be carefully weighted in terms of risks and benefits. In Table 3 we summarise the patient characteristics that can be useful for patient selection. The use of BVS in emergency bailout situations is currently not supported. Also BVS implantation in patients on oral anticoagulants should be avoided because of the need of long-term DAPT (≥3 years) due to the risk of very late ScT.

**Table 3:** Patient selection

Optimal	Patient with good life expectancy (i. e. >5 years)	Age <70 years or Age 70–80 with a maximum of 1 of: severe renal failure or dialysis, DM, BMI >40 or LVEF <40%, stroke, PAD or COPD
No potential benefit to be expected	Patient with limited life expectancy (i. e. <2–3 years)	Cardiogenic shock, severe heart failure (EF <30%), dialysis
Avoid	No use in emergency bail-out situations Patients on oral anticoagulants	–

*BMI* body mass index, *COPD* obstructive pulmonary disease, *DM* diabetes mellitus, *LVEF* left ventricular ejection fraction, *PAD* peripheral artery disease

## Technical considerations for BVS implantation

We want to stress that an optimal BVS implantation technique will be of paramount importance for obtaining good long-term clinical results [24]. Lesion preparation is especially important, as, before inflation, the initial scaffold diameter is quite large (1.4 mm) which is related to the specific scaffold-related folding characteristics of the Absorb BVS. Therefore, highly calcified or tortuous lesions or lesions with a high degree of angulation can be quite challenging for BVS implantation. However, with extensive lesion pre-dilatation using increasing balloon sizes, even calcified lesions can be successfully treated with BVS, although special care has to be paid to a good implantation technique. In summary, we advise the ‘P-S-P’ implantation technique:

1. ‘Prepare the lesion’ aggressively using adequate predilatation (1:1 vessel-to-balloon ratio), We caution not to implant BVS in lesions were predilatation balloons do not fully expand.
2. ‘Size the vessel/scaffold correctly’. On-line QCA or preferably invasive imaging (intravascular ultrasound [IVUS] and optical coherence tomography [OCT]) is advisable in general and indispensible in small vessels (reference vessel diameter <3 mm or using a 2.5 mm BVS scaffold). Evidently, undersizing has to be avoided and never implant if the RVD is below 2.5 mm (measured by invasive imaging).
3. ‘Post-dilate’ the scaffold with a properly sized non-compliant balloon to avoid underexpansion (using a non-compliant balloon with a diameter at least equal to and preferably 0.5 mm larger than the RVD using high inflation pressures [18–20 atm]).

Also, be aware of the expansion limits of the implanted BVS as overexpansion of the scaffold can potentially lead to scaffold fractures (maximal 0.5 mm larger than scaffold diameter). The effect of a BVS specific implantation strategy was deduced from a large registry covering four hospitals and >1300 patients. After implementation of a specific BVS implantation protocol, incidence of ScT was significantly reduced (3.3% vs. 1.0% at 12 months for suboptimal vs. optimal, respectively) [26]. In this retrospective analysis failure to achieve a final minimal lumen diameter of 2.5 mm for the small design (nominal 2.5 mm or 3.0 mm devices) or 2.9 mm for the larger design (nominal 3.5 mm devices) was an important factor in the occurrence of ScT.

To avoid BVS malapposition, correct scaffold sizing based on a reliable assessment of vessel dimensions is a second important issue. Invasive imaging modalities, such as IVUS and OCT, can be of great value in providing accurate morphometry for estimating vessel diameter and lesion length in circumstances where angiographic assessment is ambiguous. OCT is particularly suited to visualise the apposition of scaffold struts to the vessel wall and can guide BVS optimisation [27]. Before implantation, OCT is indicated to predetermine lesion characteristics, such as vessel diameter, lesion length and the amount of calcification, to estimate the optimal scaffold length and to identify the optimal proximal and distal landing zones. The importance of appropriate vessel sizing is becoming increasingly clear, and, as already mentioned, BVS implantation in vessels with a diameter of less than 2.25 mm on quantitative coronary imaging (comparable with a diameter of less than 2.5 mm on visual estimation) should be avoided because of substantially worse clinical outcomes and an elevated risk of ScT [4]. OCT after scaffold implantation can be a helpful tool to guide postdilatation of the scaffold with properly sized non-compliant balloons to optimise strut apposition, taking into account the expansion limit of 0.5 mm for the Absorb BVS.

## Antiplatelet therapy

DAPT has been the basis for minimising stent thrombosis for many years. Current guidelines for antiplatelet therapy advise DAPT for 6–12 months in stable angina patients receiving DES [28]. For ACS patients, based on the European Society of Cardiology non-STEMI and STEMI guidelines a minimum of 12 months of DAPT is advised, preferably with prasugrel or ticagrelor [29, 30]. More recent publications suggest that for a low-risk population and when using second generation DES, DAPT duration might be shortened to 6 months [31, 32]. With the longer follow-up period of larger registries and RCTs very late ScT has emerged as a major problem. Timewise this might be related to the resorption process of the BVS. Although in preclinical settings this has never been noticed as a problem, the current hypothesis is related to the expected degradation and disintegration of devices without full embedment in the vessel wall. Areas of malapposed, disintegrating struts might become instable and trigger thrombosis. This might be an additional risk factor to other already identified risk factors for very late stent thrombosis.

The Dual Antiplatelet Therapy (DAPT) study demonstrated that in patients who tolerated DAPT after DES implantation for 12 months, without a bleeding complication, DAPT up to 30 months, as compared with aspirin therapy alone, significantly reduced the risks of stent thrombosis and major adverse cardiovascular and cerebrovascular events but was associated with an increased risk of bleeding [33]. Within this very large (*n* = 11,648) randomised study with 348 ischaemic events, multiple risk factors were identified ([34]; Table 4) demonstrating the multifactorial process of stent thrombosis, one of which was the use of paclitaxel-eluting stents (with thick struts). Finally, a model was developed balancing the increased bleeding risk of prolonged DAPT duration with the reduction in ischaemic events (DAPT score).

**Table 4:** Predictors of myocardial infarction or stent thrombosis 12–30 months post-PCI in the DAPT study

Predictors of MI or ST	HR (95% CI)	*P* value
MI at presentation	1.65 (1.31–2.07)	<0.001
Prior PCI or prior myocardial infarction	1.79 (1.43–2.23)	<0.001
History of CHF or LVEF <30%	1.88 (1.35–2.62)	<0.001
Vein graft stent	1.75 (1.13–2.73)	0.01
Stent diameter <3 mm	1.61 (1.30–1.99)	<0.001
Paclitaxel-eluting stent	1.57 (1.26–1.97)	<0.001
Diabetes mellitus	1.38 (1.10–1.72)	0.01
Peripheral arterial disease	1.49 (1.05–2.13)	0.03
Hypertension	1.37 (1.03–1.82)	0.03
Renal insufficiency/failure	1.55 (1.03–2.32)	0.04

*CHF* congestive heart failure, *LVEF* left ventricular ejection fraction, *MI* myocardial infarction, *PCI* percutaneous coronary intervention, *ST* stent thrombosis

Recently, the PEGASUS-TIMI 54 trial [35] evaluated long-term therapy with ticagrelor in addition to aspirin, in patients with a history of spontaneous MI occurring 1–3 years prior to randomisation. The PEGASUS study concluded that ticagrelor significantly reduced the risk of MACE as compared with placebo. Recently, two meta-analyses [36, 37] showed that DAPT beyond 1 year among stabilised high-risk patients with prior MI decreased ischaemic events at the cost of an increase in major bleeding. Udell et al. showed that prolonged DAPT >1 year could reduce the rate of stent thrombosis [36].

For the Absorb BVS a minimum of 6 months DAPT is required per protocol (ABSORB-EXTEND, ABSORB II), and the majority of patients in most studies received DAPT for 12 months. Because of signals of a higher occurrence of early as well as late, and even very late stent thrombosis (beyond 1 year), currently and until further confirmation, the best advice is to prescribe DAPT for a minimum of 3 years for BVS Absorb implanted patients in a tailored approach including all known risk factors for very late stent thrombosis and predictors of increased bleeding risk [22]. In all ACS patients and possibly also in patients with more complex coronary lesions, there is a preference for the use of the more potent P2Y12 inhibitors, such as ticagrelor or prasugrel. As the prolongation of DAPT therapy from 1 to 3 years could prove to be harmful in specific patient subgroups, certainly those with a higher bleeding tendency and especially for patients already on oral anticoagulants, it is recommended to avoid implantation of the Absorb BVS in patients with a strict indication for oral anticoagulation.

## Conclusions

The new 2017 recommendations downgrade and limit the use of current BVS to experienced centres within dedicated registries using the updated implantation protocol and advise the prolonged usage of DAPT in patients at high risk of ischaemic events. Patient should be well informed on the potential risks of the therapy and the only hypothetical long-term benefit. This recommendation is based on recent evidence from large randomised trials that implantation of BVS is associated with increased risk of adverse events, particularly increased risk of ScT and MI. In the near future the COMPARE ABSORB trial will report additional data regarding the appropriateness of BVS for PCI for specific lesion and patient subsets.
